# EPR Properties of Concentrated NdVO_4_ Single Crystal System

**DOI:** 10.1007/s00723-015-0659-2

**Published:** 2015-03-18

**Authors:** S. M. Kaczmarek, H. Fuks, M. Berkowski, M. Głowacki, B. Bojanowski

**Affiliations:** 1 Institute of Physics, Faculty of Mechanical Engineering and Mechatronics, West Pomeranian University of Technology in Szczecin, Al. Piastow 17, 70-310 Szczecin, Poland; 2 Institute of Physics, Polish Academy of Sciences, Al. Lotników 32/46, 02-668 Warsaw, Poland

## Abstract

Single crystals of NdVO_4_ were grown by the Czochralski method under ambient pressure in a nitrogen atmosphere. Obtained crystals were transparent with strong violet coloring. Temperature and angular dependences of electron paramagnetic resonance (EPR) spectra of the samples in the 3–103 K temperature range were analyzed applying Dyson like lineshape typically used for concentrated magnetic system. EPR-NMR program was used to find local symmetry and spin-Hamiltonian parameters of neodymium ions.

## Introduction

Rare-earth orthovanadates are birefringent crystals and meet all necessary technical specifications to be a very prospective material for fiber optical communication systems [1–4]. They are also interesting due to their unusual magnetic characteristics and useful luminescent properties [5]. Nd^3+^:YVO_4_ is one of the most promising laser hosts for micro- and diode-pumped solid-state lasers that exhibit a multisite character [5–7]. The rare-earth vanadates and YVO_4_ crystalize in tetragonal zircon-type structure and belong to the tetragonal space group *I4*_*1*_/*amd*, *Z* = 4. In this structure the vanadium atom is tetrahedrally coordinated while the trivalent Y(Nd) cation is coordinated by eight oxygen atoms forming a bidisphenoid. NdVO_4_ is a semiconductor, *E*_g_ = 2.86 eV [8]. Rare-earth ions occupy undistorted sites (US) with local symmetry of *D*_*2d*_ and also distorted ones (DS) with lower symmetry [6, 7, 9–11].

Electron paramagnetic resonance (EPR) is a sensitive tool to study local environment around the probe ion and to estimate spin-Hamiltonian parameters of rare-earth ions in host crystals. But it is worth to remember that the spin Hamiltonian parameters in diluted systems and concentrated ones usually significantly differ from each other [6, 7]. Many papers were devoted to local sites symmetry description in YVO_4_ slightly doped with neodymium ions [1, 2, 5–7], but a few only to concentrated NdVO_4_ single crystals. The latter, however, did not apply EPR technique [9, 10]. In the paper we focus our attention on the concentrated NdVO_4_ medium to describe its local symmetry and magnetic properties using EPR spectroscopy.

## Experimental

Single crystals of NdVO_4_ were grown by the Czochralski method in an inductively heated iridium crucible of 40 × 40 mm and with a passive afterheater. Starting materials with 4 N purity were heated at 1000 °C (Nd_2_O_3_) or at 300 °C (V_2_O_5_) for 6 h before weighing, mixing and melting. Due to evaporation of vanadium from the melt during crystal growth process [12] composition of starting oxides was 1 mol.% shifted towards higher concentration of V_2_O_5_. Single crystals with 20 mm in diameter were grown on iridium 2 mm rod with pulling rate of 3–4 mm/h and a rotation of 4–6 rpm. Crystals were grown under ambient pressure in a nitrogen atmosphere. During crystallization strong tendency to spiral growth was observed. In spite of lack of crystal seed most crystals started to grow along direction close to *c*-axis with *a*- and *b*-planes (cleavage planes) on the side. Obtained single crystals were transparent with strong violet coloring.

The samples used for EPR purposes have been shaped of 2.5 × 2.5 × 3.5 mm^3^ parallelepipeds with planes perpendicular to crystallographic axes. So our laboratory axes system (LAS) is coincident with crystallographic axes system (CAS). The EPR spectra were recorded on a conventional X-band Bruker ELEXSYS E 500 CW-spectrometer operating at 9.5 GHz with a 100 kHz magnetic field modulation equipped with the standard helium gas flow system. The first crystal absorption spectra derivative was recorded as a function of the applied magnetic field. To determine the site symmetry and localization of the paramagnetic probe in REVO_4_ lattice, the angular dependencies of RE^3+^ have been measured. All registered EPR spectra of the investigated samples were simulated using the EPR-NMR computer program to study the spin-Hamiltonian parameters [13].

## Results

Applying XRD technique, we have registered the X-ray rocking curve of NdVO_4_ single crystal for the symmetrical (400) reflections. Full width at half maximum, FWHM = 0.042°. It indicates good crystallographic quality of the investigated sample.

**Fig. 1:**
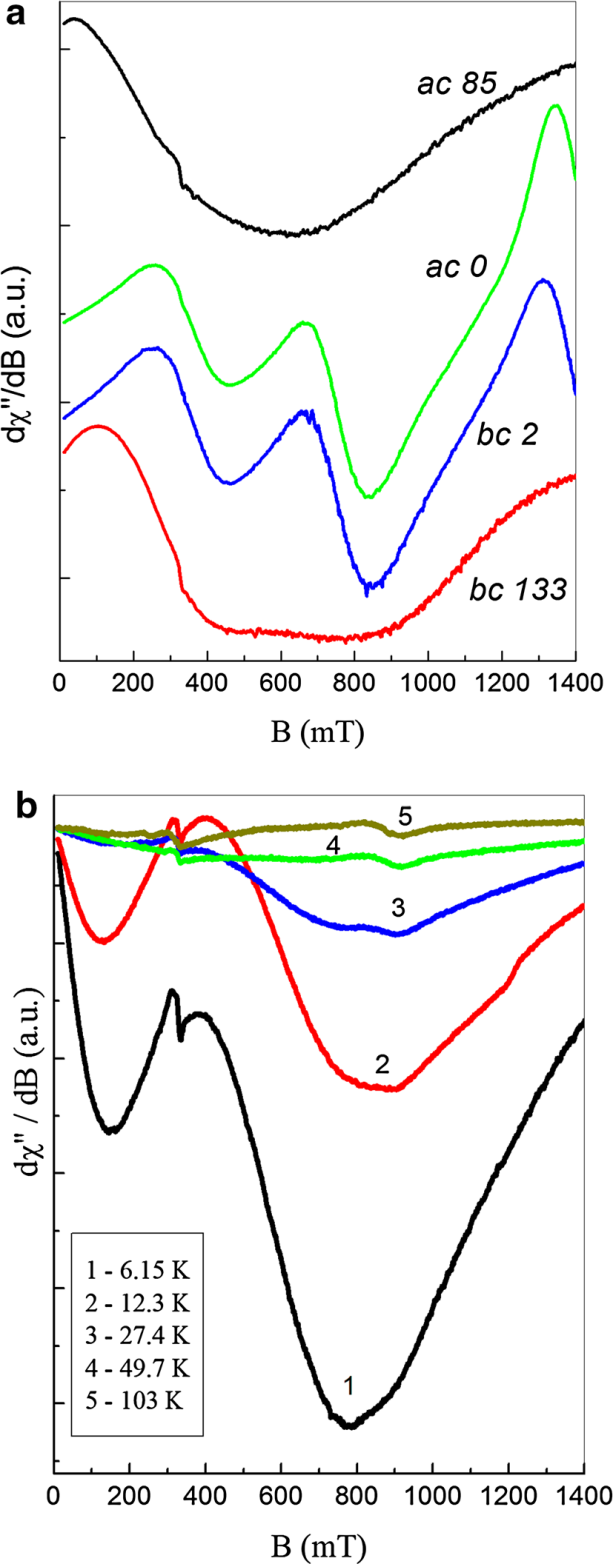
**a** Several EPR spectra registered at selected position of the NdVO_4_ single crystal in *ac*- and *bc*-planes at a temperature of 7 K. *Numbers* inside the *picture* represent theta angle between magnetic field and *c*-axis directions. **b** EPR spectra registered for several temperatures in *ab*-plane.* Narrow line* centered at 340 mT has an external origin. Signals did not changed with a change of rotation angle

Well oriented NdVO_4_ single crystal was investigated by using EPR technique. X-band resonance spectra were detected at temperature range 3–103 K, at three perpendicular planes of magnetic field operation. Resonance signal consisted of three, more or less resolved wide lines (Fig. 1a), observed in a whole magnetic field range. These wide lines may be a superposition of several different paramagnetic centers. The origin of these lines can be assigned to neodymium magnetic ions present in the regular NdVO_4_. Moreover, besides at regular sites, there can be present also neodymium ions at interstitials. As it was found in [6, 7], Nd^3+^ ions are inhomogeneously distributed in tetragonal *D*_*2d*_ symmetry sites, as isolated ions, “shallow clusters” and pairs. Additionally, during doping with increasing Nd^3+^ concentration to YVO_4_, an increase in a content of paramagnetic defects—V^4+^ ions is observed. These ions could affect the EPR spectrum of NdVO_4_ single crystal. We have observed V^4+^ ions in the EPR spectrum of HoVO_4_ single crystal [14].

Nd^3+^ ions possessing *4f* ^*3*^ electronic configuration are characterized by ^*4*^ *I*_*9/2*_ term. When these ions are affected by low symmetry crystal field, this term is split into five Kramers doublets, among which only the lowest doublet with an effective spin *S* = 1/2 is populated, due to energy intervals [4, 15, 16].

All the above mentioned papers concern mainly to materials weakly doped by neodymium, where the resonance signal is narrow and hyperfine structure of odd isotopes is clearly observed as a satellite components around the central line. But a large concentration of the Nd^3+^ ions leads to overlapping of the EPR signal and disappearance of the hyperfine lines [17]. In the paper, in addition to isolated Nd^3+^ ions, magnetic pairs of neodymium ions with an effective spin *S* = 1/2 of each component were taken into account.

In Fig. 1a several low-temperature EPR spectra registered at selected positions of the crystals rotation angle in *ac*- and *bc*-planes are presented. In Fig. 1b the temperature dependence of the EPR spectra registered for several temperatures in the *ab*-plane and arbitrary rotation angle are shown. As one can see, EPR spectra registered in *ab*-plane (Fig. 1b) show decrease in the signal intensity as a function of temperature. A line centered at about 340 nm is due to pipe glass used for EPR measurements purpose.

Usually, EPR absorption signal is described by a first derivative of Lorentzian line. Such a model, being generally valid in a low concentrated magnetic system, should be modified if we analyze dense magnetic system. The shapes of the EPR spectra could be described in this case by a function proposed by Dyson [18], where the EPR line is broadened due to a dispersion phenomenon. It is valid especially in conducting materials, such as metals, semimetals, semiconductors [19]. NdVO_4_ single crystal is a semiconductor. So our supposition about Dysonian-like EPR lineshape is qualified. The function adapted to our case could be written as:

1 $$\frac{{{\text{d}}\chi ''}}{{{\text{d}}B}} = \frac{{\alpha \,\Delta B^{2} - 2\,\Delta B(B - B_{\text{r}} ) - \alpha (B - B_{\text{r}} )^{2} }}{{[(B - B_{\text{r}} )^{2} + \Delta B^{2} ]^{2} }}$$

where, *α* is the dispersion factor, Δ*B*, is the width of a resonance line, *B*_r_, is the position of a resonance line.

**Fig. 2:**
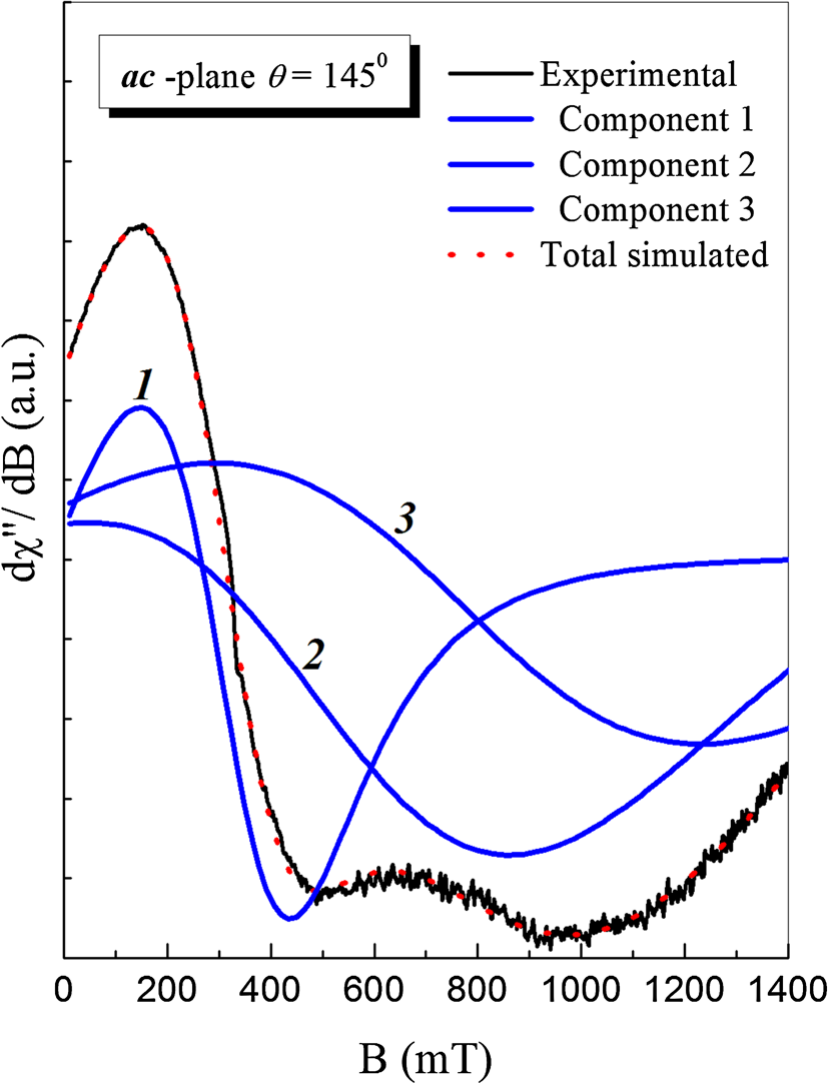
Decomposition of the EPR resonance spectrum measured at 7 K for three Dyson-type components according to Eq. (1)

We have performed decomposition of the EPR lines to at least three different Dyson-like components. Possibility of distinguishing of three different resonance lines (see Fig. 2) is connected probably with an existence of at least three different magnetic centers in the investigated system.

Among all the three spectroscopic components detected in the NdVO_4_ single crystal, the line located at a magnetic field below 400 mT revealed shape and intensity, both being independent on rotation angle. Contrary to this, both two higher located components revealed huge deviation with rotation angle change (Fig. 1a). The above properties allowed us to conclude, that the low-field line and two higher field lines have different origin. We assigned the line centered at low magnetic fields to isolated (single) neodymium magnetic centers, whereas two components observed at higher magnetic fields to more complex magnetic systems, i.e. magnetic pairs.

**Fig. 3:**
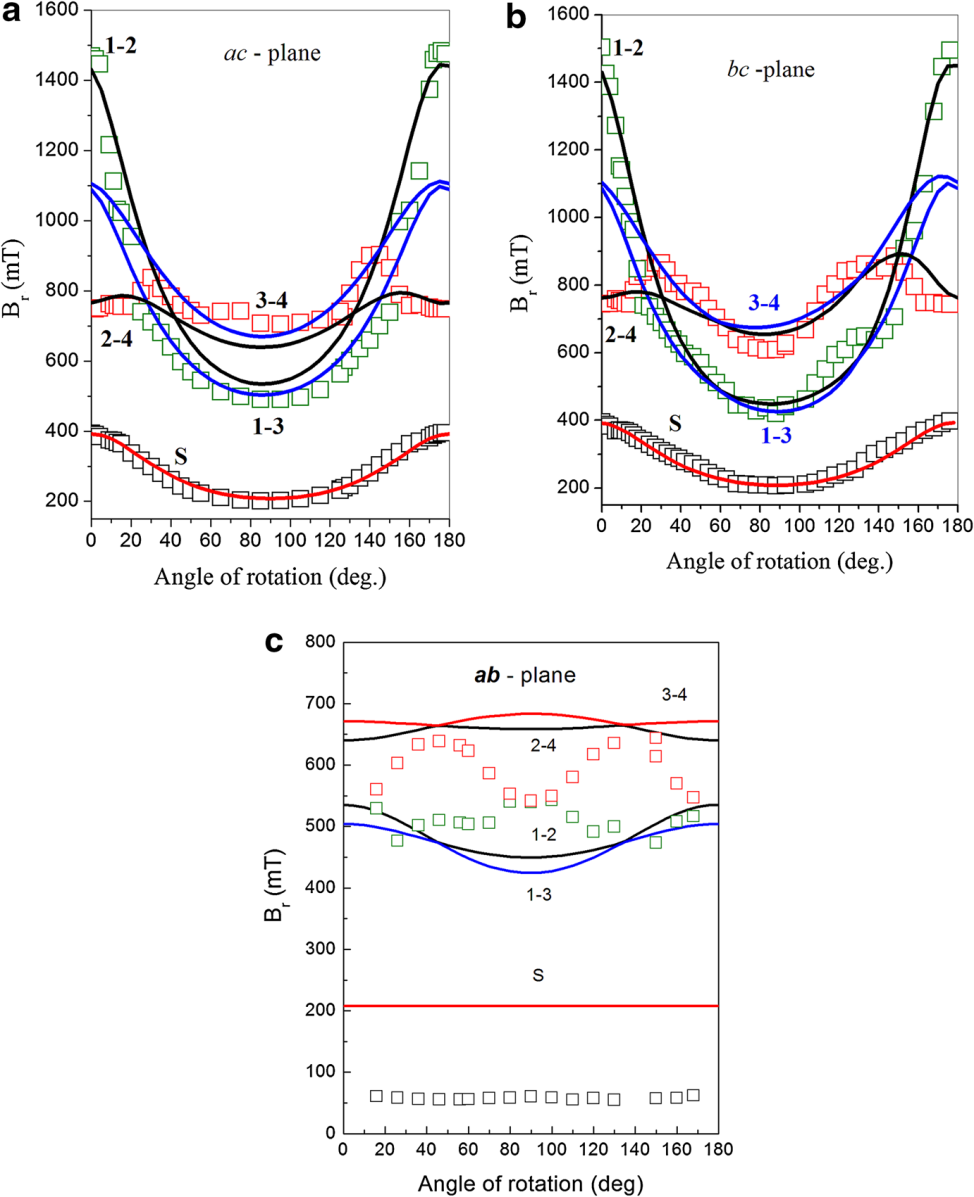
Positions of the three spectral components as a function of theta angle (*squares*): **a** *ac*-plane, **b** *bc*-plane and **c** *ab*-plane. Measurements were performed for *T* = 3.5, 4.5 and 6 K, respectively. For **a** and **b** the values 0 and 180° mean that the magnetic field, *B*, was operated in a *c*-direction, whereas 90° in both planes *ac* and *bc *represents *B* vector aligned in *a*- and *b*-directions, respectively. Label *S* mark a signal assigned to isolated (single) magnetic ions. *Solid lines* were obtained with using Eq. (2). *Inside* the pictures we have shown labels of transitions inside a pair

Applying Eq. (1) to our EPR spectra allows us to estimate position of all three components of analyzed EPR signal as a function of a crystal rotation angle. Full angular dependences of the EPR lines positions (roadmaps) obtained for all crystal rotation planes (*bc, ac* and *ab*) are presented in Fig. 3 (open squares). The angle presented in horizontal axis is defined as azimuthal, theta angle, between magnetic field and *c*-experimental axis direction in Fig. 3a, b, whereas in Fig. 3c it is phi angle. Phi angle is defined as an angle between magnetic field vector projection on *ab* plane and *a* direction. As both roadmaps in *bc*- and *ac*-planes looks very similar, one can conclude that in the third plane, marked in this case as *ab*-plane, a resonance signal should be almost constant vs. rotation angle (see Fig. 3c). Such conclusion seems to be consistent with those reported in [4]. Indeed, our EPR spectra of NdVO_4_ crystal registered in *ab*-plane are only weakly affected by crystal rotation. Unfortunately, calculated position and width of the lines doesn’t fit well with results obtained in *ac*- and *bc*-planes. This curious discrepancy is assigned probably to existence of some external lines (V^4+^ and/or interstitial defects) in experimental data registered in *ab*-plane, that lead to significant changes of resulting EPR spectra [14].

EPR line positions presented in Fig. 3 (squares) were fitted by using EPR-NMR program [13]. For lower part of presented pattern, in which an EPR signal is expected to arise from isolated neodymium centers, we adopted a simple model including only Zeeman term with effective spin *S* = 1/2. As could be seen, the results of calculations presented in Fig. 3 as solid lines (low fields, marked by letter *S*), are very satisfying.

From the other hand, for components located at higher magnetic fields, we supposed that paired Nd–Nd centers are responsible for the signal. Keeping in mind an effective value *S* = 1/2 of each neodymium magnetic ion, we took into account two different physical regimes: strong and weak exchange energy inside the pair. Strong exchange regime should lead to an effective spin value of the pair *S* = 1, where spin Hamiltonian contains zero-field splitting term interaction: *SDS*, described elsewhere. Including calculated line positions and their relative intensity, such attempts failed in our case.

More satisfying results we obtained with regime of magnetic pairs, where in basic model an effective spin maintains values of *S*_1_ = *S*_2_ = 1/2 for each component. In this case a spin Hamiltonian has the following form:

2 $$H_{\text{s}} = \mu_{\text{B}} BgS + S_{1} JS_{2}$$

where, the first term is a Zeeman term interaction and the second one represents exchange interaction among *S*_1_ and *S*_2_ spins of the magnetic pair. The *J* is exchange matrix, containing both: isotropic and anisotropic exchange interactions and dipole–dipole interactions (see for example [17]).

The results of calculation, involving Eq. (2), are presented in Fig. 3 as solid lines in upper part of pattern. They seem to be quite satisfying. Including transition probabilities, one can accept that at angles around 0° and 180° only 1–2 and 2–4 transitions could be observed, whereas at position around 90° most probable to be seen are 1–3 and 3–4 transitions.

We have calculated *g*-matrix parameters. For Nd–Nd pairs, they are described mainly by the following diagonal values: *g*_*aa*_ = 1.130, *g*_*bb*_ = 1.207, *g*_*cc*_ = 0.610 and *J*_*aa*_ = 750 G, *J*_*bb*_ = 1400 G, *J*_*cc*_ = −1050 G, whereas for isolated magnetic centers we obtained: *g*_*aa*_ = *g*_*bb*_ = 3.250, *g*_*cc*_ = 1.720.

**Fig. 4:**
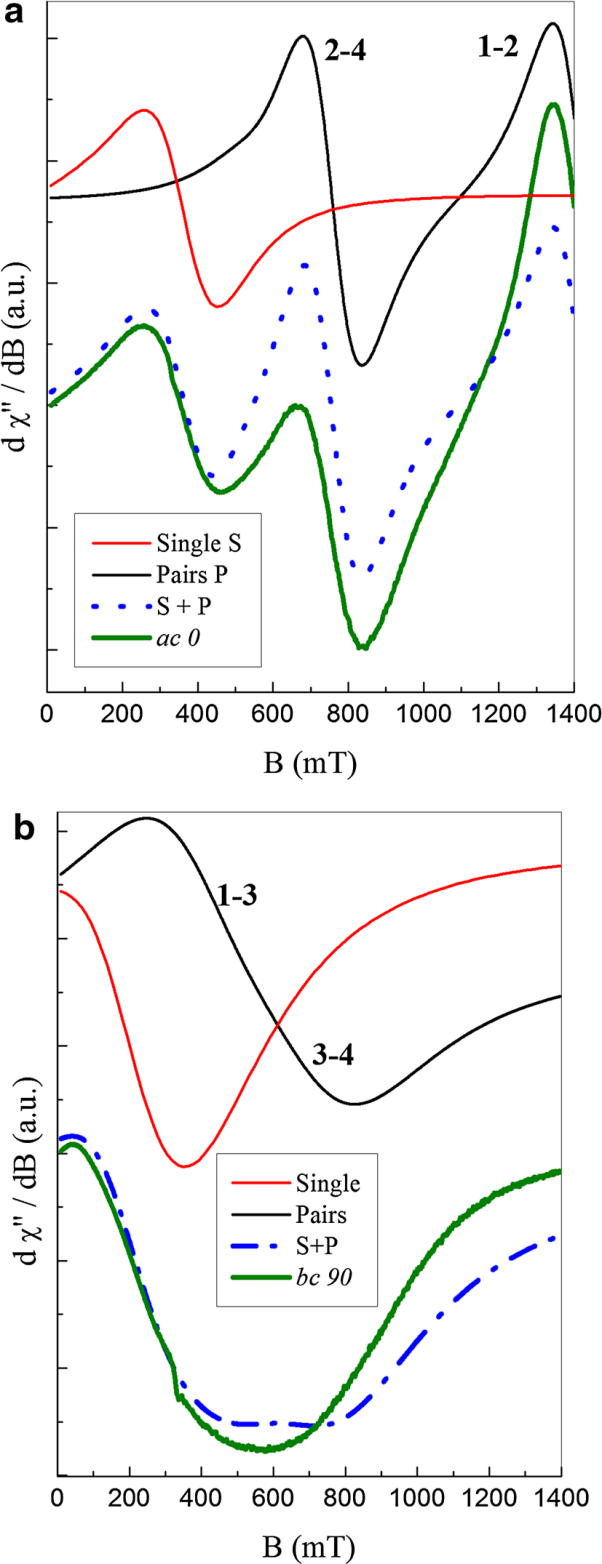
Comparison between simulated spectra built as a sum of isolated and paired magnetic centers (*dotted lines*) and two arbitrary chosen experimental spectra described as: *ac 0* and *bc 90*: **a** *ac*-plane, **b** *bc*-plane

Detailed analysis including an angle position and relative intensity of all components of the resonance signal is presented in Fig. 4. In the figure we have shown calculations of an EPR signal shape, supposing its origin as a sum of a single and paired neodymium magnetic centers (lines *S* + *P*) for two different crystal orientations: perpendicular to *ac* and *bc* planes, and two different magnetic field directions with respect to *c* **-**axis: 0° and 90°. Due to a large width of the lines we took into account both circular components of the lines including negative part of magnetic resonance field, *B*_r,_ for the line observed below 400 mT. The results presented in Fig. 4 were obtained applying Eq. (1) for the line *S*, whereas for the paired signal a simple Lorentzian line shape was taken into account.

The calculated lines are not perfectly fitted to experimental lines: *ac* 0 and *bc* 90, but generally reflects sensibility of the applied model. One of a reason of the discrepancy could originate from omitting the asymmetric part of *H*_s_ in Eq. (2). But a second possible reason could arise from the model of line shape.

**Fig. 5:**
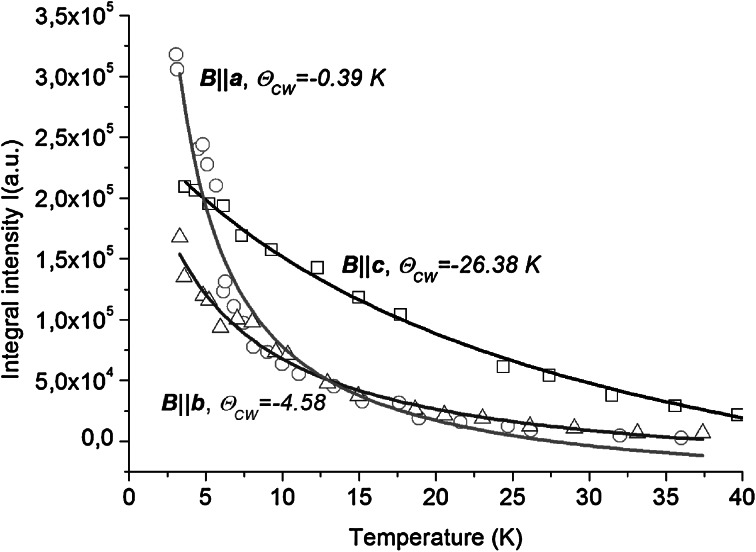
Temperature evolution of the integral intensity of EPR lines calculated for all three directions: magnetic field B||*a*, B||*b* and B||*c*

Figure 5 presents temperature evolution of an overall EPR integral intensity, *I*, for all three planes: *ab*, *ac* and *bc*. As could be seen, concentrated NdVO_4_ magnetic system fulfills the Curie–Weiss relation *I* = *C*/(*T* − *θ*_CW_) − *I*_o_.

Detailed calculation allowed us to obtain characteristic parameters *θ*_CW_ = −4.58 K for *ac*-plane, −0.39 K for *bc*-plane and −26.38 K for *ab*-plane, respectively. As was previously mentioned, the observed signal in *ab*-plane contains additionally some external lines (V^4+^ and/or interstitial Nd^3+^ defects ~100 mT), so obtained value of *θ*_CW_ in this case is uncertain. But taking into consideration only results obtained for *ac*- and *bc*-planes one can conclude that the negative value of Curie–Weiss temperature indicates the dominating antiferromagnetic interactions between Nd^3+^ magnetic centers. Solid lines plotted in Fig. 5 represent the classical Curie–Weiss law, but to improve the fitting, some diamagnetic component (*I*_o_) was additionally taken into account. The origin of this component may serve, e.g., diamagnetic V^4+^ pairs with very short mutual distance [20].

**Fig. 6:**
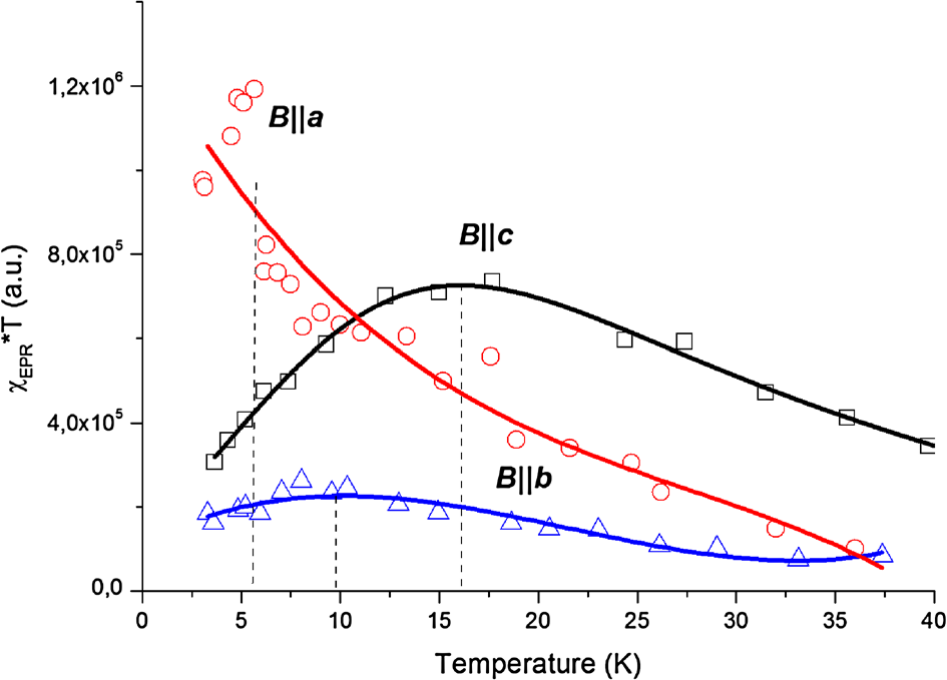
Temperature evolution of a χ_EPR_*T product calculated for all three directions. *Solid lines* are guides for eyes

Significant discrepancy between *θ*_CW_ values calculated for *ac*- and *bc*-planes indicates the anisotropy of magnetic properties of NdVO_4_. It is clearly shown also in Fig. 6, where the dependence of the product of the integral intensity and temperature is plotted vs. temperature for each plane. It is proportional usually to a square of magnetic moment.

## Discussion

YVO_4_ crystals exhibit generally a multisite character. Two types of Nd^3+^ sites were determined by EPR technique in the YVO_4_:Nd crystal: (1) undistorted (US) sites with tetragonal *D*_*2d*_ symmetry, 99.9 %; (2) distorted (DS) sites with lower symmetry (*C*_*2v*_ or *D*_*2*_) [4]. It was found that the distortion of this DS site most probably originates from a defect in the neighboring of V^5+^ sites. The principal signal in the YVO_4_ doped with Nd^3+^ diluted system is attributed to Nd^3+^ in a tetragonal site with *D*_*2d*_ point symmetry [4]. In our case, by analogy to diluted YVO_4_:Nd system, lower line observed in Fig. 3, extending between 200–400 mT, may be assigned to isolated Nd^3+^ ions located at the US sites. Indeed, for this site *g* matrix values are characterized by the following relation *g*_*aa*_ = *g*_*bb*_, indicating on axial symmetry of this magnetic center.

From the other hand, two lines observed in this figure above 400 mT may be assigned to Nd–Nd pairs. Calculated values of *g* and *J* matrices indicate on a weak anisotropy of above mentioned magnetic centers. It is observed as a small asymmetry of the patterns presented in Fig. 3.

The presence of complex magnetic centers, observed in EPR spectra, is assigned to different distances between neodymium sites in such dense magnetic system and to V^4+^ ions presence.

## Conclusions

Single crystals of NdVO_4_ were grown by the Czochralski method. They were optically transparent with strong violet coloring. EPR spectra of the samples in the 3–30 K temperature range, registered in three *ac*-*, bc*- and *ab*-planes, show three broad EPR lines. The lines were analyzed applying fitting with three Dyson-like components. This kind of fitting is usually used for concentrated magnetic systems. EPR-NMR program was applied to find local symmetry and spin Hamiltonian parameters of neodymium ions, for which an effective spin of *S* = 1/2 was adopted. Among the lines, the lower magnetic field line we assigned to Nd^3+^ ions located at isolated undistorted sites. The high magnetic field lines we proposed to be attributed to pairs of Nd^3+^ ions. From the temperature dependence of EPR lines measured in all three planes it results that NdVO_4_ single crystal reveals antiferromagnetic kind of interactions and strong anisotropy of the magnetic properties.
